# Key Amino Acid Residues of Ankyrin-Sensitive Phosphatidylethanolamine/Phosphatidylcholine-Lipid Binding Site of βI-Spectrin

**DOI:** 10.1371/journal.pone.0021538

**Published:** 2011-06-28

**Authors:** Marcin Wolny, Michał Grzybek, Ewa Bok, Anna Chorzalska, Marc Lenoir, Aleksander Czogalla, Klaudia Adamczyk, Adam Kolondra, Witold Diakowski, Michael Overduin, Aleksander F. Sikorski

**Affiliations:** 1 Laboratory of Cytobiochemistry, Biotechnology Faculty, University of Wrocław, Wrocław, Poland; 2 Max Planck Institute of Molecular Cell Biology and Genetics, Dresden, Germany; 3 Department of Molecular Biology, University of Zielona Góra, Zielona Góra, Poland; 4 Henry Wellcome Building for Biomolecular NMR Spectroscopy, School of Cancer Sciences, University of Birmingham, Birmingham, United Kingdom; University of Cambridge, United Kingdom

## Abstract

It was shown previously that an ankyrin-sensitive, phosphatidylethanolamine/phosphatidylcholine (PE/PC) binding site maps to the N-terminal part of the ankyrin-binding domain of β-spectrin (ankBDn). Here we have identified the amino acid residues within this domain which are responsible for recognizing monolayers and bilayers composed of PE/PC mixtures. *In vitro* binding studies revealed that a quadruple mutant with substituted hydrophobic residues W1771, L1775, M1778 and W1779 not only failed to effectively bind PE/PC, but its residual PE/PC-binding activity was insensitive to inhibition with ankyrin. Structure prediction and analysis, supported by *in vitro* experiments, suggests that “opening” of the coiled-coil structure underlies the mechanism of this interaction. Experiments on red blood cells and HeLa cells supported the conclusions derived from the model and *in vitro* lipid-protein interaction results, and showed the potential physiological role of this binding. We postulate that direct interactions between spectrin ankBDn and PE-rich domains play an important role in stabilizing the structure of the spectrin-based membrane skeleton.

## Introduction

The shape and deformability of erythrocytes are maintained by the presence of the membrane skeleton, which associates with the plasma membrane through a combination of protein-protein and protein-lipid interactions. The main player in formation of this network is spectrin, which attaches to the plasma membrane primarily through ankyrin and other binding partners [1], [2], [3], [4], [5]. In addition, the ability of spectrin to bind to phospholipids of the membrane inner leaflet remains of considerable interest. Studies of spectrin-lipid interactions, dating from the early nineteen seventies, have revealed that spectrin repeats possess lipid-binding properties [6]. Several binding sites for phosphatidylserine (PS)-rich domains are evident on both α- and β-spectrin subunits, and supporting roles for these sites in spectrin-4.1R protein and spectrin-ankyrin binding have been suggested [7], [8], [9], [10], [11]. The importance of PS asymmetry for maintaining the mechanical properties of the erythrocyte membrane was documented by Manno et al. [12]. Apart from its PS-binding ability, spectrin also binds to PE-rich domains, as shown by others [13] and by us [14], [15]. The PE lipid-binding site was found to be located within the ankyrin-binding domain (ankBDn), encompassing the 14th and 15th segment [16] of β-spectrin and situated within the N-terminal 38 amino acid residues of this domain [17], [18]. We showed previously that binding of erythroid spectrin to PE/PC mixtures, both phospholipid vesicles and monolayers, was competitively inhibited by ankyrin, a feature that was only limited in the case of PS binding by erythroid spectrin [14]. This suggested the possibility of a regulatory mechanism based on inhibition of spectrin-PE binding by ankyrin.

Further structural studies using site-directed spin labeling technique suggested the presence of a possible hydrophobic surface that could be responsible for the interaction with lipids [19]. The recently published structure of the ankyrin-binding domain and its complex with ankyrin revealed that the putative lipid-binding site would be buried inside the coiled-coil structure and a major structural rearrangement of the triple helical bundle would be needed for its exposure [20], [21]. Indeed, as was reported by Czogalla et al. [22] during the interactions of ankBDn with PE/PC liposomes some structural changes in the mutual alignment of the helices were observed and an “opening” of the structure was proposed.

The present study aimed to further explore the possibility that the previously proposed hydrophobic patch is the main site for interactions with PE. To address this issue we constructed a series of point mutations inside the lipid-binding part of the ankyrin-binding domain. Using various techniques we measured the associations of these mutants with PE/PC mono- and bilayers. Our results indicate that residues W1771, L1775, M1778 and W1779 are directly responsible for lipid-binding activity within the ankyrin-binding domain of erythroid β-spectrin. On the basis of structural data reported by us [22] and others [20], [21] as well as our biochemical analyses we provide a consistent model of this interaction and possibly its inhibition by ankyrin. Furthermore, we present data obtained from studies on resealed erythrocyte ghosts and HeLa cells that show morphological changes dependent on the presence of the intact, mutation-free, N-terminal lipid-binding site within the ankyrin-binding domain of β-spectrin.

Considering that both PE and PS are asymmetrically distributed in the membrane and that erythrocyte membrane holds more PE than PS [23], the ability of spectrin to interact with PE in an ankyrin-dependent manner could be physiologically important. It has been shown that a normal spectrin skeleton is present in ankyrin-deficient mouse erythrocytes, demonstrating the potential of spectrin to form stable attachment sites to the plasma membrane [24]. As suggested previously by Sikorski and Białkowska [25], the major attachment site would be the PE-binding domain. Here we provide further evidence that the lipid-binding feature of ankBDn is of functional importance, based on observations of GFP-tagged ankBDn and its mutants in cells.

## Results

### Expression, purification and characterization of the ankyrin-binding domain of β-spectrin and its mutants

**Figure 1:**
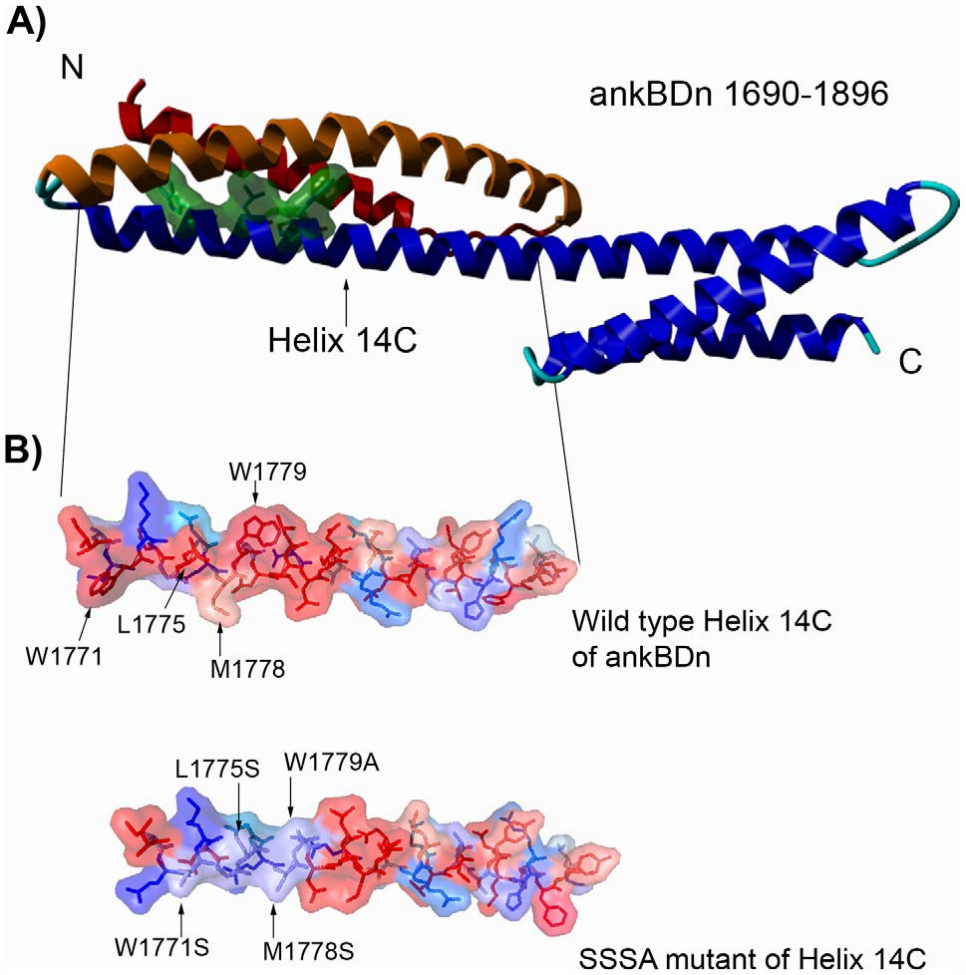
Models of ankyrin-binding domain. A) Ankyrin-binding domain, with residues W1771, L1775, M1778, W1779 marked in green. Helix 14A is shown in red, helix 14B is colored orange. B) Hydrophobicity analysis of a 38 amino acid fragment of ankyrin-binding domain (ankBDn) and its mutant (SSSA) by 3D-mol software. Colors represent hydrophobicity of amino acid residues: deep red, hydrophobic; and deep blue, hydrophilic. Arrows indicate exchanged residues.

**Figure 2:**
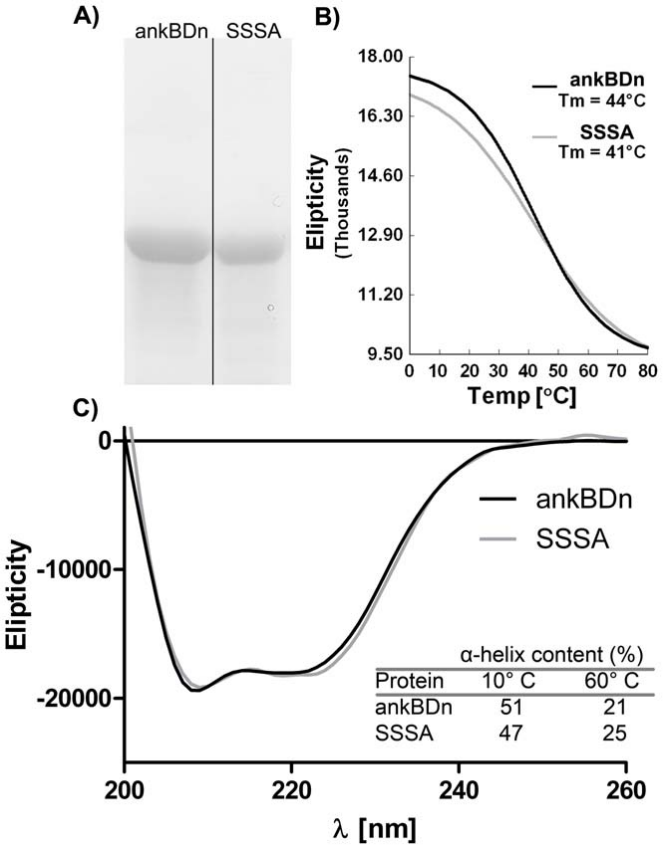
Purity and secondary structure of wild-type and mutant protein. A) Purified proteins. Electrophoresis was carried out in 10% polyacrylamide gel in the presence of 0.1% SDS. Gels were stained with Coomassie Blue. Arrow indicates ∼42 kDa molecular mass. B) Melting curves of proteins shown in B). The T_m_ was determined by second derivative calculation. C) CD spectra of recombinant ankyrin-binding domain (ankBDn) and quadruple mutant SSSA at 20°C. Inset: comparison of α-helix content calculated from CD spectra obtained at indicated temperature.

Our previous results indicated that the ankyrin-sensitive PE/PC-binding site of β-spectrin was limited to the 38 amino acid residues in the N-terminal fragment of the 14C helix of the ankyrin-binding domain [18]. Figure 1A shows this fragment and its placement in the helical context of ankBDn. The localization of these residues as presented in this figure suggests the formation of a hydrophobic patch, which is consistent with the data obtained by Czogalla et al. [19], [22]. Below we describe experiments to identify amino acid residues crucial for binding PE/PC mono- and bilayers. We constructed a series of mutants that contained substitutions in the region of residues 1771–1779 (mutants are named for the mutated residues they contain) and 1782–1786 (names starting as Mut...), as these are the most hydrophobic regions in the N-terminal fragment of the helix 14C of the ankyrin-binding domain (1768–1805). The primary intent of these substitutions was to reduce the exposed hydrophobic surface of this region of spectrin in order to test whether this was key to ankBDn-lipid interactions. We also avoided creating “patches” of serine residues; therefore, in some mutants, W1779 was substituted by alanine. The difference in the hydrophobicity index of S and A residues is negligible [26]. Indeed, the introduced substitutions affected the hydrophobicity parameters [26] of the protein (Fig. 1B) but, as observed by the CD spectra (examples are shown in Fig. 2B,C), had little effect on the secondary structure of the domain in terms of either the helical structure content at 10°C or the melting temperature. This indicates that the observed changes in binding activities of the mutants stem from local and not global structural changes.

### PE/PC-monolayer binding activity of ankBDn and its mutants

**Figure 3:**
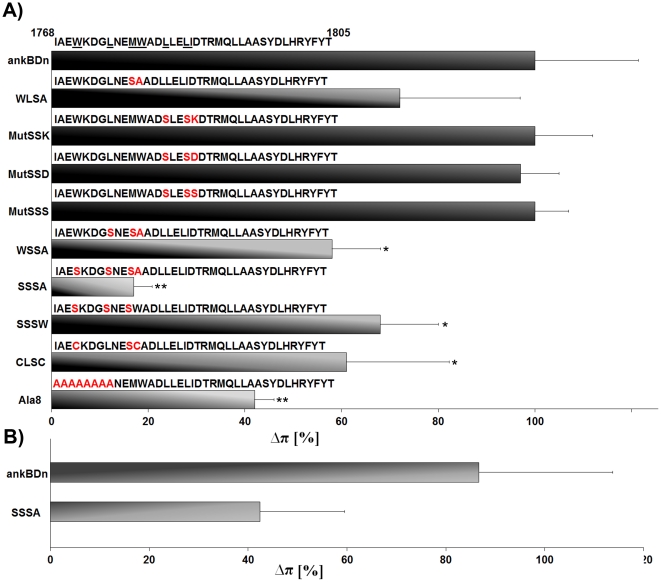
Interaction of recombinant ankyrin-binding domain (ankBDn) and its mutants with PE/PC lipid monolayer. A) Interaction of ankBDn and its mutants with PE/PC 3∶2 (w/w) monolayer. The first 38 amino acid residues of ankBDn are shown; substitutions are marked in red. The ankBDn plateau level is defined as 100% PE/PC binding activity and is equal to ∼2.5 mN/m. Plateau levels were obtained at a protein concentration of ∼25–35 nM. Higher plateau levels indicate increased penetration of the lipid monolayer by protein. Average values were calculated from at least three measurements. Significant differences from the plateau value obtained for wild-type ankBDn are marked (Student's t test): * p<0.05; ** p<0.0001. Details in “Materials and Methods”. B) Interaction of ankBDn and its quadruple mutant SSSA with PS/PC 3∶2 (w/w) monolayer. Interaction of ankBDn with PE/PC monolayer was defined as 100%.

For basic characterization of full-length ankBDn and its mutants we used a monolayer assay, which provides a convenient tool for the detection of protein-lipid interactions, as changes in the surface pressure indicate differences in the level of interactions between a protein and the lipid monolayer. In our experiments we used a “loose” monolayer of PE/PC at a 3∶2 (w/w) ratio at initial surface pressures of 8–12 mN/m [27]. Although it is far from the 30–35 mN/m pressures typical for natural membranes, these conditions give important indications for lipid-protein interaction, as was previously shown [18], [28], [29]. The combined results are shown in Figure 3A. The plateau levels of *Δ*π for the mutants were normalized using the level of wild-type ankBDn (2–3 mN/m) as 100%. Also shown are the sequences of the N-terminal 38 residues of wild-type ankBDn and the mutants.

The first of these mutants to be tested was WLSA, which was a double mutation M1778S/W1779A (see Fig. S1A). The monolayer assay showed that WLSA protein retained ∼75% of its lipid-binding ability as compared to ankBDn, suggesting that the major contribution to lipid binding was located more to the N- or C-terminus of the WLSA mutant. To address the latter possibility, we generated three mutants, called “Mut…” to underline the fact that they contained substitutions within the hydrophobic stretch of residues located in the middle of the 38-residue lipid-binding region of ankBDn: MutSSK, L1782S/L1785S/I1786K; MutSSD, L1782S/L1785S/I1786D; and MutSSS, L1782S/L1785S/I1786S (Fig. S1B). The results indicated that these three mutant proteins had the same lipid-binding capacity as the wild-type protein (Fig. 3A).

To investigate the key lipid-binding determinants on the N-terminal portion of the ankBDn we created WSSA, which contained the three mutations L1775S/M1778S/W1779A (Fig. S1C), and SSSA, which was a quadruple mutant, W1771S/L1775S/M1778S/W1779A (Fig. 1B). Both of these mutants exhibited decreased lipid-binding activity in the monolayer assay, and the decrease was dependent on the number of mutations. WSSA retained 60–75% of lipid-binding activity, while SSSA, the quadruple mutant, retained only 20% of lipid-binding activity as compared to ankBDn. To specifically assess the role of tryptophan residues, a second triple mutant, SSSW, W1771S/L1775S/M1778S (Fig. S1D) was constructed. As measured in the monolayer system, the observed decrease in *Δ*π was comparable for both mutants, reaching ∼65–75%. A final triple mutant, CLSC, W1771C/M1778S/W1779C (Fig. S1E), was constructed with the intent to test how PE/PC binding would be affected when the bulky tryptophan residues (W1771 and W1779) were exchanged with smaller, but basically hydrophobic cysteines. It was found that CLSC behaved comparably to the other triple mutants tested. The lipid-binding ability was retained at the level of 60–70%, as measured by the plateau level in the monolayer assay.

Finally the last of our constructs was “Ala8” in which the 8 amino acid residues (residues 1768–1775) were substituted by alanine to confirm our previous data obtained from truncated mutants [18]. This substitution should not compromise the helical structure of this part of the polypeptide chain as alanine residues prefer an α-helical arrangement and should mimic well the N-terminal 8 residue deletion mutant [18]. In the case of this mutant (1768–1775A, see Fig. S1F), its ability to affect the PE/PC monolayer surface pressure was decreased by more than 50% as compared to wild-type ankBDn (Fig. 3A), indicating that, although significantly lowered, the ability of Ala8 to interact with PE/PC monolayers was retained. We interpret the data shown in Fig. 3 to indicate that lipid binding is largely mediated by residues within the first 12 amino acids of ankBDn.

In addition to binding PE, spectrin also binds to PS [9], although with less sensitivity to ankyrin. Consequently, we also measured interactions of ankBDn and the SSSA mutant with PS/PC monolayers. The results presented in the Figure 3B indicate that while the PS-binding ability of wild-type ankBDn is very similar to that observed for PE/PC monolayers, the SSSA mutant retains ∼50% PS/PC binding compared to the abovementioned ∼20% of PE/PC monolayer binding. The difference is not unexpected as positively charged residues which are present within the studied region (for example K1772) probably contribute to PS-binding activity [30]. It seems sufficient to quench PE binding just by substitution of the hydrophobic residues. The positively charged residues may still stabilize PS-binding activity of this site.

### Characterization of PE/PC-liposome binding ability of ankBDn and its mutants

**Figure 4:**
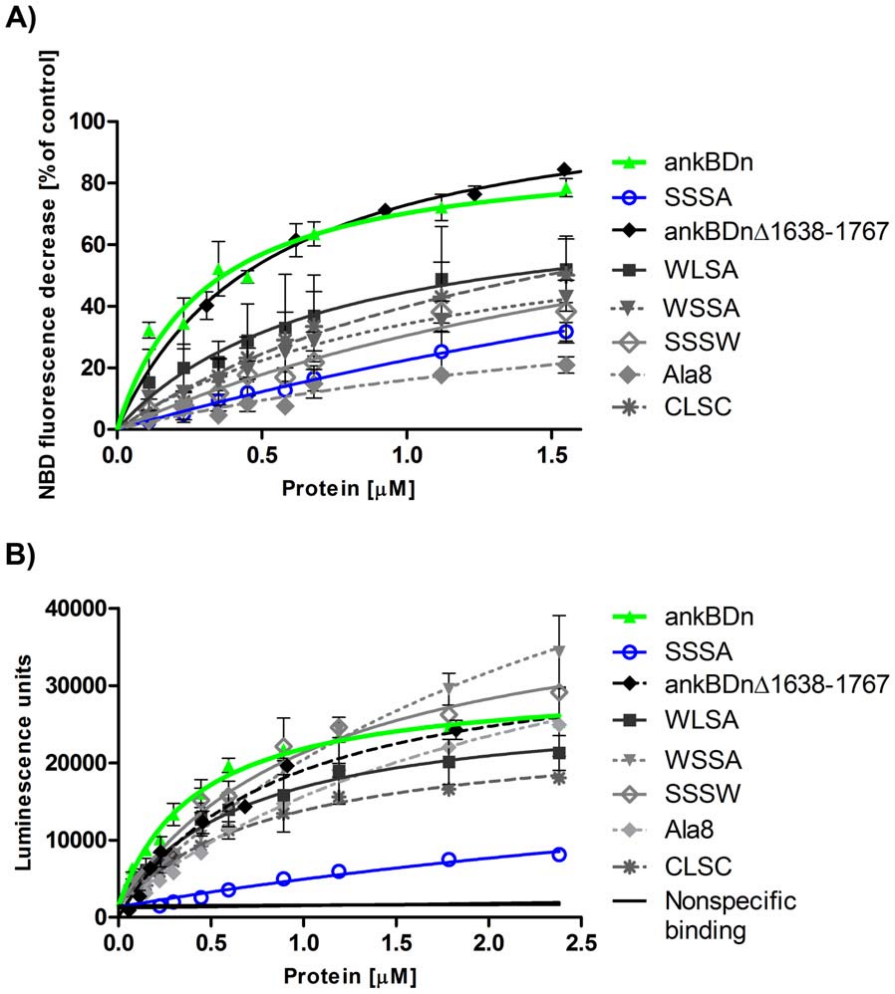
FRET-based assay (A) and EIA-based assay (B) for PE/PC binding of ankBDn and its mutants. A) Differences in binding affinity of ankBDn and its mutant shown by FRET-based assay. FRET between TRITC-labeled proteins and PE/PC liposomes containing NBD-PE was detected as a decrease in NBD-lipid fluorescence emission intensity upon protein binding to the liposomes. As a control of nonspecific binding, TRITC-labeled IgG was used. Average values were calculated from at least three independent experiments. Error bars indicate standard deviation. B): Binding of recombinant proteins by PE/PC liposomes. EIA assay: a multi-well plate was coated with PE/PC liposomes and incubated with the indicated proteins. Anti-His-Tag antibodies were used for protein detection and anti-mouse-STAG antibodies were used for electrochemiluminescence reaction. Thermally denatured ankBDn was used as a negative control. Average values calculated from at least three independent experiments. Error bars indicate standard deviation.

The monolayer technique is useful for screening for lipid-binding properties of proteins but the data it provides are unsatisfactory for obtaining the kinetic parameters of the interaction. To further address the role of substituted residues in the lipid-binding process, and to acquire kinetic data concerning these interactions, we carried out several assays, including a pelleting assay, FRET-based assay, and EIA. As expected from the monolayer experiments, the lowest affinity for PE/PC vesicles seen in the FRET-based assay was observed for the SSSA mutant (K_D_>5 µM). The calculated K_D_ of the SSSA mutant was increased at least 10-fold compared to ankBDn (K_D_ = 0.31±0.07 µM; ±SE) (Fig. 4A). The double mutant, WLSA, exhibited moderate affinity toward liposomes, as the measured equilibrium dissociation constant was in the submicromolar range (0.71±0.34 µM; ±SE). The triple mutants SSSW (K_D_>2 µM), WSSA (K_D_ = 1.1±0.24 µM; ±SE), and CLSC (K_D_>1 µM) exhibited significant decreases in affinity towards PE/PC liposomes. Also, the K_D_ for Ala8 was several-fold higher than the K_D_ for ankBDn, confirming our observation from the monolayer assay that the major contribution to lipid binding in ankBDn comes from the hydrophobic patch within the first 12 N-terminal amino acid residues. We also performed experiments with a shorter version of ankBDn with an intact putative lipid binding site (ankBDn*Δ*1638–1767). The obtained dissociation constant value for this protein (K_D_ = 0.53±0.06 µM; ±SE) was similar to that of ankBDn, which further confirmed that the PE/PC-binding site is localized on helix 14C.

A second quantitative approach was undertaken to cross-check the above observations. EIA was performed as a modified ELISA assay, which allows testing of the binding of unlabeled proteins to immobilized liposomes (for details see Materials and Methods) (Fig. 4B and Table 1). The values calculated for ankBDn and the mutants show significant consistency with the measurements presented above, with only slight variation for the SSSW and WSSA triple mutants (Fig. 4B). As can also be seen, the measurements obtained by this method strengthen the observation that the quadruple mutation in SSSA leads to almost complete loss of binding to PE/PC vesicles. We also performed a pelleting assay with maleimide rhodamine labeled proteins and FAT PE/PC liposomes. The data (Fig. S2 and Table 1) show significant differences in the affinity of ankBDn and mutants of ankBDn for PE/PC liposomes. The SSSA mutant failed to reach saturation over the range of concentrations studied. The difference between binding curves of SSSA occurring in FRET *versus* pelleting assays could be due to extensive low-affinity, nonspecific binding of SSSA protein to FAT liposomes. In conclusion, the data from the quantitative binding assays demonstrated that mutants having at least one of the native amino acid residues W1771, L1775, M1778, or W1779 retained some lipid-binding activity compared to the quadruple mutant, SSSA.

**Table 1:** Summary of dissociation constants (K_D_) and B_max_ values of the interaction of the recombinant ankyrin-binding domain and its mutants with PE/PC liposomes obtained from the FRET-based analysis (Fig. 4A), EIA (Fig. 4B), and pelleting assays (Fig. S2).

	FRET analysis		EIA		Pelleting assay	
Protein	K_D_ [µM] ±SE	B_max_±SE	K_D_ [µM] ±SE	B_max_±SE	K_D_ [µM] ±SE	B_max_±SE
ankBDn	0.31±0.07	91.7±6.9	0.43±0.05	29562±1205	0.34±0.09	3.6±0.4
SSSA	>5	-	>5	-	>5	-
WLSA	0.71±0.34	76.5±17.5	0.7±0.17	25284±2553	0.78±0.3	6.7±1.5
CLSC	>1	106.9±31.2	0.81±0.06	23016±714	>1	6.5±0.8
WSSA	1.14±0.24	73.4±8.8	>2	70101±7493	>1	4.5±0.9
Ala8	>2	54.3±17.2	>2	46209±3146	>2	8.2±2.0
SSSW	>2	105.6±38.2	1.03±0.17	39479±3136	>2	6.8±1.1
ankBDnΔ 1638–1767	0.53±0.06	111.3±3.9	0.85±0.09	35121±1544	0.71±0.3	4.4±0.8

### Competition between recombinant proteins and native spectrin for liposome binding

**Figure 5:**
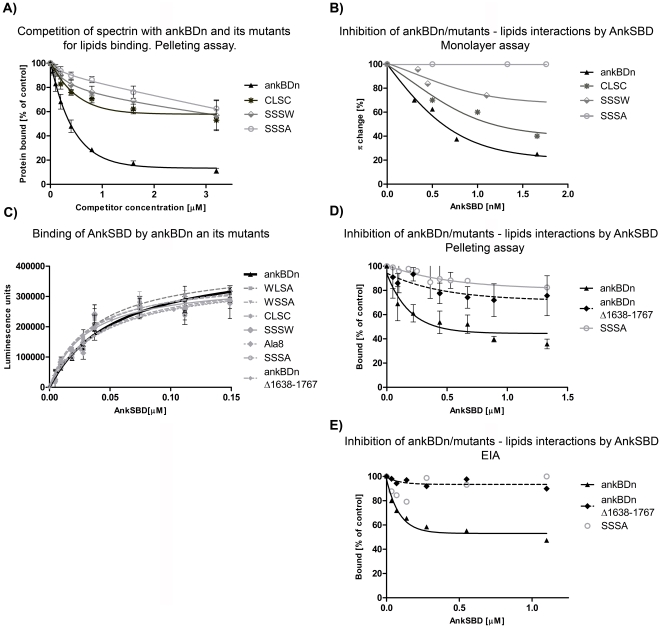
Inhibition assays and binding of ankSBD to ankBDn and its mutants. A) Inhibition of spectrin-PE/PC liposome binding by ankBDn and its mutants. Fluorescently labeled erythrocyte spectrin (50 nM) was incubated with FAT PE/PC liposomes in the test buffer (see Materials and Methods) at room temperature in the absence (100% binding) or presence of the indicated concentrations of recombinant proteins. B) Inhibition of binding of ankBDn and its mutants to PE/PC monolayers by ankSBD. Recombinant ankBDn or its mutants were preincubated in appropriate proportions with the recombinant ankSBD to obtain the indicated concentrations of ankyrin protein and concentrations of spectrin proteins inducing the Δπ changes below the plateau value. The data points represent average values of three independent experiments with an average variation<10%. C) Binding of ankSBD to ankBDn and its mutants. Data obtained by EIA assay indicate similar affinities of all studied proteins for binding ankSBD. K_D_ values were in the range 27–55 nM. D) Pelleting assay results of inhibition of binding of ankBDn and its mutants to PE/PC liposomes by ankSBD. Recombinant, fluorescently labeled ankBDn or its mutants were preincubated with FAT PE/PC liposomes at room temperature in the absence (100% binding) or presence of the recombinant ankSBD at indicated concentrations. E) EIA results of inhibition of binding of ankBDn and its mutants to PE/PC liposomes by ankSBD.

To determine whether the introduced mutations would affect the ability of ankBDn to compete for lipids with purified erythrocyte spectrin, the binding of fluorescently labeled spectrin by phospholipid vesicles in the presence of recombinant ankBDn or its mutants, SSSW, SSSA, and CLSC, was carried out. The wild-type recombinant ankBDn effectively inhibited binding of labeled spectrin (50 nM) to PE/PC liposomes, but the SSSW and SSSA mutants of ankBDn were poor competitors (Fig. 5A). The CLSC mutant also failed to effectively compete with native spectrin for liposome binding, although it exhibited a level of competition slightly higher than that of the “serine” mutants. As a control, unlabeled spectrin was used as the inhibitor. An effective level of inhibition, reaching 80% at a concentration of 750 nM unlabeled spectrin, was observed when the concentration of labeled spectrin was 50 nM (data not shown).

These results show that recombinant ankBDn bound liposomes and effectively competed with purified unmodified erythroid spectrin. Introducing mutations in the N-terminal fragment of ankBDn abolished this ability.

### Ankyrin (ankSBD) specifically disrupts interaction of ankBDn with PE/PC lipid surfaces

To test whether the residual PE/PC-binding activity of the ankBDn mutants was sensitive to ankyrin, we performed inhibition experiments based on the monolayer assay. We used the same panel of mutants presented above, and instead of native ankyrin, we used the fully active, recombinant spectrin-binding domain of erythrocyte ankyrin (ankSBD) [31]. PE/PC-ankBDn binding showed strong sensitivity to inhibition by ankSBD (Fig. 5B). The triple mutants SSSW and CLSC exhibited decreased sensitivity to inhibition by ankSBD. The residual PE/PC-binding activity of the SSSA mutant failed to show any significant sensitivity to ankyrin (Fig. 5B). K_I_ values for ankBDn, CLSC and SSSW proteins were 0.15, 0.17, and 0.2 nM, respectively (it was not possible to calculate a K_I_ for SSSA). Because the residual PE/PC-binding activity of the quadruple mutant (SSSA) was insensitive to inhibition by ankSBD, this binding is probably not determined by the hydrophobic patch within the first 12 residues.

These results suggested that the hydrophobic patch within the N-terminal 12 amino acid residues of ankBDn was also involved in the binding of ankyrin. This is consistent with the data of others [20], [21] suggesting the involvement of residues E1770, D1773, E1777, D1781 in binding of ankyrin. Therefore, mutations in this region might affect ankyrin binding. To address this issue we measured the binding of ankBDn and its mutants to ankSBD using EIA. We found that the binding curves and K_D_ values were similar for all studied proteins (∼ 27–55 nM), ruling out the possibility that the lipid-binding region of ankBDn plays a crucial role in ankyrin binding (Fig. 5C). This result indicates that the conformation of the protein constructs was close to the native form as they retained ankyrin-binding activity.

### Ankyrin inhibits opening of the coiled-coil structure of ankBDn, preventing its interaction with lipids

Ankyrin sensitivity is the main feature of ankBDn-PE/PC interactions, yet its molecular mechanism remains unknown. Crystallographic data regarding a complex of spectrin-ankyrin fragments suggest that the binding site for ankyrin is located on helices 14A and 14C of spectrin [20], [21], [32], [33]. In this work we postulate that interaction between ankBDn and lipids is based on exposure of the lipid-binding site located on helix 14C, which requires movement of helix 14A. As ankyrin may bind to both helices, it may block their movement upon lipid binding. To check this hypothesis we performed inhibition experiments in which we used, besides ankBDn and SSSA mutant, ankBDn*Δ*1638–1767 protein. This peptide lacks helices 14A/B, yet is fully active in lipid and ankyrin binding, as shown here (Fig. 4, Fig. 5C) and in our previous work [18], [34].

The obtained results confirmed that lipid binding of ankBDn is highly sensitive to inhibition by ankyrin as it is decreased by ∼60%, as shown by sedimentation and EIA assays (Fig. 5D, 5E). What is interesting, however, the lipid-binding property of ankBDn*Δ*1638–1767 protein was only slightly affected by ankyrin. These results, although somewhat different from those obtained previously [18], suggest that the majority of the ankyrin inhibitory effect probably stems from blocking of the movement of helix 14A, which prevents exposure of the lipid-binding site.

**Figure 6:**
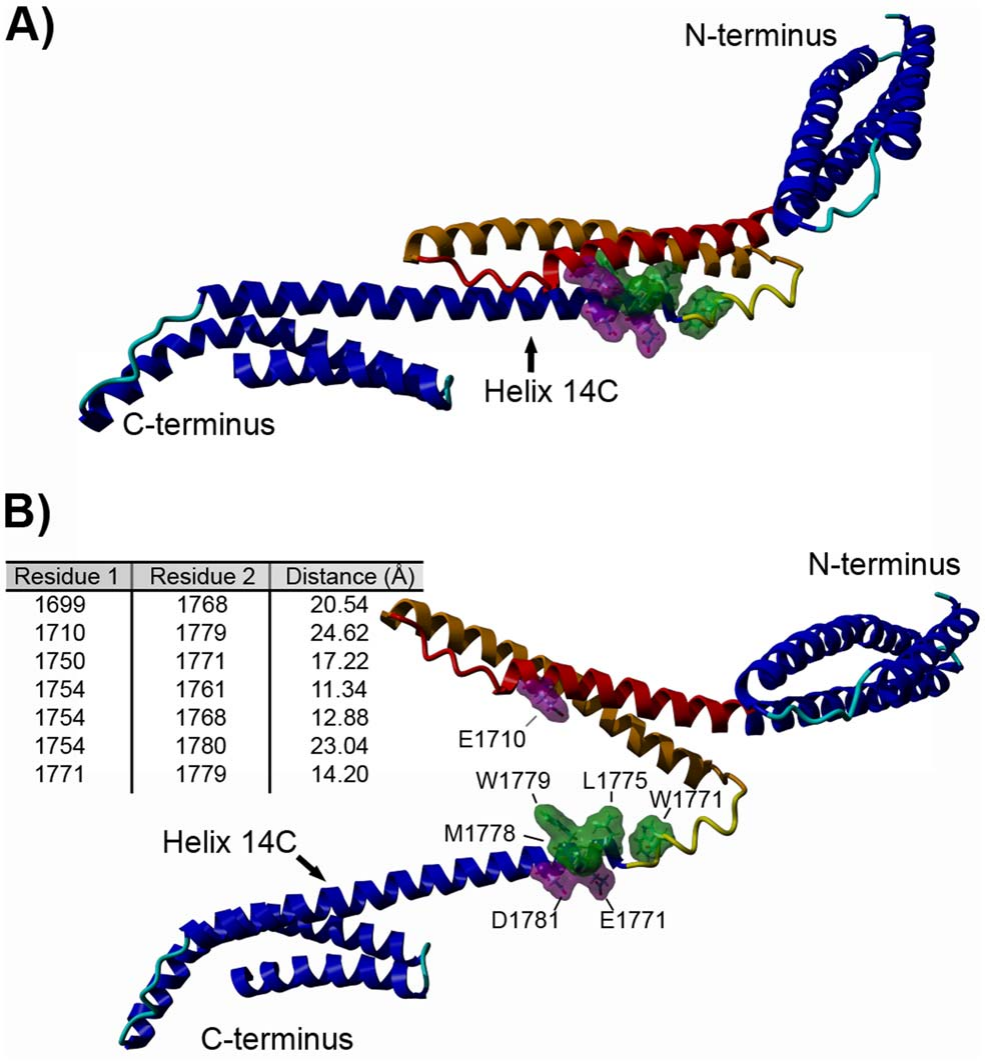
Model of ankBDn implementing 3_10_ helix in N-terminus of helix 14C. A) “Closed” structure of ankBDn. B) The model of the “open” conformation of ankBDn which is detected upon lipid binding. Table shows distances between pairs of α carbons between proximal amino acid residues in the energy-minimized model of spectrin repeat (ankBD) in the presence of PE:PC liposomes. Amino acid residues responsible for lipid binding (W1771, L1775, M1778, W1779) are marked in green; those marked in violet are residues engaged in ankyrin binding located in close proximity of lipid-binding site. The 3_10_ helix element is marked in yellow, helix 14A is marked in red, helix 14B is marked in orange.

This hypothesis is supported by the model of ankBDn upon lipid binding (Fig. 6), which suggests that helix 14A movement uncovers amino acid residues engaged in lipid binding. This model also explains the mechanism of inhibition by ankyrin, as residues that take part in interaction with ankyrin are located on both 14A and 14C helices (Fig. 6A, 6B). Ankyrin binds to both helices, preventing their separation, and in consequence inhibits interaction with lipids.

### Effects of ankBDn and its mutants on morphology of resealed erythrocyte ghosts

Spectrin-based membrane skeleton is particularly important in erythrocytes as they lack other skeletal/scaffold structures. In the work of Chorzalska et al. [35] it was shown that wild-type ankBDn caused changes in the morphology and membrane properties of erythrocyte ghosts. We decided to check what effect, in comparison to the effect caused by ankBDn, mutants of ankBDn that partially (CLSC) or completely (SSSA) lack lipid-binding ability would have on erythrocyte ghosts.

**Figure 7:**
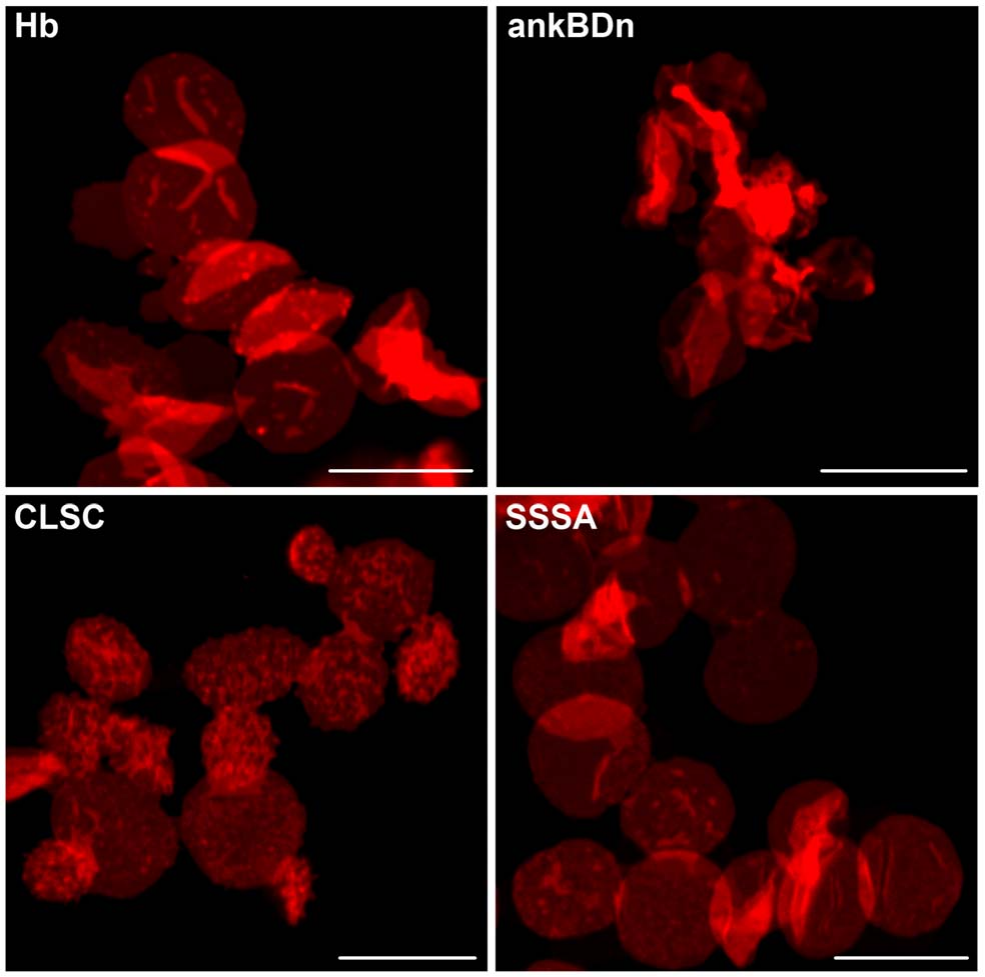
The effect of resealing erythrocyte ghosts in the presence of ankBDn and its mutants. Hemoglobin (∼1 mg/ml) or bacterially expressed and purified ankBDn and its mutants, CLSC and SSSA (∼1 mg/ml), were present in the resealing buffer. Resealed erythrocyte ghosts were labeled with DID. Ghosts loaded with wild-type ankBDn show the most extensive shape change while those loaded with SSSA mutant resemble control ghosts loaded with hemoglobin. For details see Materials and Methods. Scale bars: 10 µm.

Ghosts resealed in the presence of ankBDn exhibited enormous deformations, whereas ghosts resealed in the presence of the CLSC mutant of ankBDn exhibited formation of echinocytes (Fig. 7). An interesting, although not unexpected, observation was a lack of morphological changes in ghosts resealed in the presence of the SSSA mutant in comparison to control ghosts resealed in the presence of hemoglobin, which was used as a carrier protein to stabilize the recombinant proteins against precipitation. Also, some release of erythrocyte spectrin (Fig. S3) during the resealing incubation was observed in the presence of ankBDn but was not observed during resealing in the presence of mutant proteins (Fig. S3).

### Effect of ankBDn and its mutants on membrane skeleton of transiently transfected HeLa cells

In recent studies by Chorzalska et al. [35] it appeared that the spectrin-based skeleton aggregation in HeLa cells correlated with the presence of the N-terminal part of the ankBDn molecule. Moreover, the co-aggregation of overexpressed ankBDn-conjugated construct and endogenous spectrin [28] was accompanied by aggregation of transmembrane proteins that are known to interact with membrane skeletal structures via ankyrin, such as Na^+^/K^+^ATP-ase, L1 CAM and IP3 receptor protein. However, co-aggregation of ankBDn did not affect the actin skeleton or distribution of cadherins [35]. The effects on cells were consistent with the postulate that aggregates were connected to the membrane via ankyrin. In this study we tested the effect of mutations in ankBDn on its ability to affect the morphology of spectrin-based skeletal structures of HeLa cells. We observed co-distribution of ankBDn or its mutants, SSSA and CLSC, with Na^+^/K^+^ATP-ase, which indicates that our observations pertain to the same events observed in our previous analysis of the truncated mutants [28], [35].

**Figure 8:**
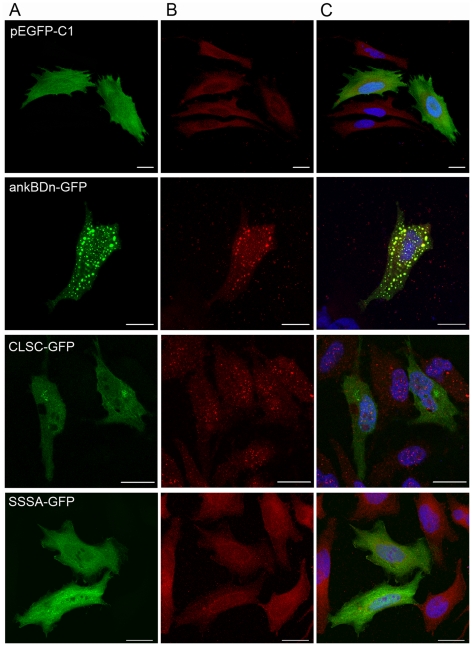
Transient overexpression of ankBDn and mutants in HeLa cells. A) Overexpression of GFP-conjugated constructs; B) Immunofluorescent staining with anti-Na^+^/K^+^-ATPase antibodies; C) Overlay. Distribution pattern of Na^+^/K^+^-ATPase is changed in cells transiently overexpressing ankBDn and CLSC constructs. This is especially visible in cells overexpressing ankBDn, where GFP constructs colocalize with Na^+^/K+-ATPase. GFP plasmid was used as control. Scale bars: 20 µm.

HeLa cells were transfected with the reporter vector pEGFP-C1, carrying the subcloned wild-type ankBDn or its mutants. Empty pEGFP-C1 reporter plasmid was used as a control. Overexpression of the ankBDn-GFP construct resulted in its aggregation, which can be seen as a punctate pattern. The presence of spectrin in the aggregates was revealed with anti-spectrin antibodies depleted of activity for ankBDn (data not shown, [28]). The co-distribution of Na^+^/K^+^ATP-ase and ankBDn aggregates is obvious in cells overexpressing wild-type ankBDn and less obvious in cells overexpressing CLSC mutant. Cells overexpressing the SSSA-GFP construct and control cells were indistinguishable (Fig. 8).

We also assessed possible endosomal localization of aggregates by immunostaining with anti-EEA1 antibodies. We found that there was no co-localization between ankBDn-GFP-spectrin aggregates and endosomes (Fig. S4A). Finally, we performed western blot analysis of transfected cells, which showed that GFP-fusion proteins remained intact in HeLa cells, i.e. no substantial proteolysis was observed (Fig. S4B).

In conclusion, these experiments on red blood cells and transiently transfected HeLa cells showed that introduction of recombinant ankBDn into those cells resulted in significant changes in their morphology, and in formation of aggregates of spectrin-based skeleton and the overexpressed protein, while introduction of recombinant proteins with substitutions of the four residues in the PE/PC-binding element failed to induce these changes. This supports the idea that lipid-binding properties of ankBDn are of physiological importance.

## Discussion

Our previous studies on interactions of spectrin with lipids revealed PE/PC-binding activity of β-spectrin. A key feature of this interaction was its sensitivity to inhibition by purified ankyrin so the disposition of the PE/PC-binding site within the ankyrin-binding domain was possible. Further studies defined a region in the N-terminal part of the ankyrin-binding domain of the β-spectrin involved in PE/PC binding [18]. In the next step of our research, presented in this work, we identified key amino acid residues responsible for lipid binding. Our approach was based on the construction of several site-directed mutants of ankBDn with different combinations of substitutions within the lipid-binding site of this domain. The aim was to introduce mutations that would perturb the hydrophobic surface in helix 14C, which appeared to be well positioned to contact the bilayer (Fig. 1B, Fig. S1).

Our experiments showed that much of the PE/PC-binding property of ankBDn is confined within 12 spectrin amino acid residues (1768–1779). This interpretation is derived largely from the observation that, compared to ankBDn, binding to the monolayers by the quadruple mutant, SSSA, was markedly decreased and that the other constructs carrying mutations within the same region exhibited reduced abilities to bind lipid mono- and bilayers. The combined results obtained from various lipid-binding assays reveal that PE/PC-binding activity resides within helix 14C of β-spectrin. Furthermore, the residues W1771, L1775, M1778, and W1779 form a hydrophobic surface on one side of the helix, which is involved in lipid/PE/PC binding. Any mutation of the hydrophobic surface in helix 14C formed by these four proximal residues led to a decrease in lipid-binding affinity. Moreover, the lipid-binding affinity was inversely related to the number of replacements of hydrophobic residues in this region of the molecule, i.e. K_D_ ankBDn<2 mutations<3 mutations<4 mutations. To quench PE/PC-binding activity all four residues should be replaced in the recombinant ankBDn. We also measured ankyrin sensitivity of ankBDn and its mutants. The fact that residual binding of the quadruple mutant, SSSA, to the PE/PC monolayer was insensitive to inhibition by ankyrin, coupled with the fact that ankyrin inhibited about 80% of ankBDn binding, suggested that the residual 20% of ankBDn binding is determined by residues that are not involved in this lipid-binding site [6]. The results of the EIA showed that all studied proteins had similar, high affinity for ankSBD, indicating that binding to ankSBD is unaffected by the introduced substitutions. We now conclude that the minimal ankyrin-dependent, PE/PC-binding motif in erythrocyte spectrin is 1771WXXXLXXMW1779.

A closer look at the crystal structure of the 14^th^ segment of spectrin shows that the lipid-binding residues are buried between helices A and C, suggesting that they are hardly accessible in this state for interaction with the bilayer. To explore the possibility of a structural arrangement of the PE-binding site we attempted to built a model of the lipid-bound ankBDn. The model was based on X-ray crystal structures of the β-spectrin lipid-free state but incorporated restraints from site-directed spin labeling data collected in the presence of PE/PC [19], [22]. The mentioned EPR studies were performed in solution and measured changes in distance between site-directed spin labeled residues on helices of the 14^th^ spectrin segment upon lipid binding. The distance between the spin centers located in helix A and C was larger than 25 Å [22], which suggested an interesting possibility for a mode of interaction of ankBDn with the lipid bilayer, in which the helices of the spectrin 14^th^ repeat partially separate from each other when interacting with lipids. The solution state model also includes an SDSL-based 3_10_ helical element between residues 1764–1773 and an unstructured conformation N-terminal to helix C.

Based on the EPR restraints, binding of PE/PC mono- and bilayers by ankBDn is predicted to result in an “open” structure with an exposed lipid-binding surface. Using the three distances measured between proximal pairs of residues and assuming no major changes in secondary structure of ankBDn, the membrane binding event causes the triple helical bundle to separate and expose the N-terminal part of helix 14C which spans the lipid-binding site. This interaction site is no longer sterically restricted, and would be free to interact with membrane phospholipids through exposed hydrophobic and basic residues.

A similar mechanism was also proposed for the interaction of dystrophin rod domains with anionic phospholipids as well as for other proteins with tryptophan residues engaged in lipid-binding activity [11], [36]. Using the above-mentioned results of our previous structural work we explored the possibility of structural arrangement of the PE/PC-binding site. A model of ankBDn based on crystal structures combined with the results of SDSL studies was constructed (Fig. 6). The EPR spin-spin distance measurements showed a possibility of the presence of 3_10_ helix in the N-terminal part of helix C of the 14^th^ segment of spectrin [19], [22]. Although this is a comparatively minor change to the crystal structure, it presents some interesting features. As shown in Fig. 6, W1771 shifts outside the triple helical bundle when the 3_10_ helix is modeled. This residue could work as an initial point of attachment of the protein to the lipid bilayer. Also the position of other hydrophobic residues shown to be engaged in lipid binding, including L1775, M1778 and W1779, further supports our proposed mechanism as they form a compact hydrophobic patch. Finally, our EPR-based model is an extrapolation from the static crystal structure, and takes into account the conformational flexibility in the N-terminal region of helix C in solution [37], [38].

Although the model presented here explains the accessibility of the lipid-binding site, its ankyrin sensitivity still remains to be addressed. The recently published crystal structure of the spectrin-ankyrin complex [20], [21], [32], [33] sheds new light on the role of ankyrin in the lipid-binding mechanism. Hydrophilic residues from region 1770–1781 of the 14C helix (E1770, D1773, E1777, D1781) as well as E1710 from the 14A helix were reported as residues forming an ankyrin-binding interface on spectrin [20], [21], [32], [33]. These residues are in close proximity to the lipid-binding site as defined by four hydrophobic residues: W1771, L1775, M1778, and W1779. This proximity may provide an answer to the question about the inhibitory effect of ankyrin on lipid binding. We suggest that the ability of ankyrin to inhibit ankBDn lipid binding is probably a result of steric interference by ankyrin, which is caused by binding the E1710 residue which blocks the “opening” of the triple helical bundle. This residue is located close to the lipid-binding site, on helix 14A, and ankyrin may prevent movement of this helix, which seems essential for PE binding. The fact that the truncated mutant ankBDn*Δ*1638–1767, which has the full lipid-binding site but lacks helices 14 A/B, is basically insensitive to ankyrin seems to positively verify the aforementioned hypothesis.

It is generally accepted that the major attachment site of spectrin to the membrane occurs through the interaction with ankyrin. To assess the potential physiological role of the lipid-binding domain we designed experiments to test the interactions in the systems that mimic the *in vivo* environment. Detailed experiments on truncated mutants have been recently published [35], so here only examples of results of experiments performed on resealed erythrocyte ghosts and HeLa cells transfected with constructs containing “wild-type” or selected ankBDn mutants are presented. Surprisingly, although ankBDn and its mutant show similar binding capacities (K_D_ and B_max_) for ankyrin (Fig. 5C), the deformation of resealed ghosts was directly dependent on the potential of these constructs to bind PE/PC (Fig. 7). The occurrence of dramatic changes in morphology of erythrocyte ghosts resealed in the presence of ankBDn, and their lack in the presence of SSSA, suggested the importance of the mutated residues in maintaining mechanical properties of the membrane and in maintaining the biconcave shape of erythrocytes. These data support the data of Chorzalska et al. [35], who showed that truncated mutants of ankBDn with greatly decreased lipid-binding activity also failed to impose changes on ghost membranes, and showed that barrier properties of erythrocyte ghosts resealed in the presence of ankBDn were reduced. In a different experiment, HeLa cells overexpressing ankBDn presented a punctate pattern and displacement of spectrin associated with changes of distribution of the integral membrane protein, Na^+^/K^+^ATPase. On the other hand, overexpression of triple or quadruple mutants produced few, if any, changes in comparison to control cells (Fig. 8). These changes cannot be attributed to ankyrin binding, but rather highlight the importance of lipid-binding properties of the ankyrin-binding domain, as demonstrated by the partial or complete loss of lipid binding in the CLSC and SSSA mutants, respectively.

Previous work by Bennett et al. [39] showed that transfection of intestinal caco-2 cells by the ankyrin-binding domain of β_G_-spectrin leads to loss and displacement of endogenous spectrin and to occurrence of a punctate pattern in cells. Here we observe similar aggregates of overexpressed ankBDn but not of its mutants, that have decreased lipid-binding properties. Moreover, co-localization with Na^+^/K^+^-ATPase and presence of spectrin in aggregates of ankBDn-GFP suggest that the spectrin-ankyrin-Na^+^/K^+^-ATPase complex remains intact in contrast to the effect of expression of the spectrin-binding domain of ankyrin, which extensively disrupts the structure of the spectrin-based skeleton [31]. Altogether, these data reveal that PE/PC binding of spectrin may be of more importance for cellular homeostasis than previously thought.

Keeping in mind that each spectrin tetramer has two ankyrin-binding domains and that in the erythrocyte membrane the number of ankyrin molecules is equal to the number of spectrin tetramers, the number of sites unoccupied by ankyrin is high. These unoccupied sites may be bound to PE/PC-rich domains. We speculate that overexpression of ankBDn, or its introduction into erythrocyte ghosts, induces detachment of spectrin from the lipid-binding sites and consequently permits its aggregation or release. The ability of recombinant ankBDn to compete with spectrin for liposomes under *in vitro* conditions, and the absence of changes in cells with overexpressed/resealed SSSA mutant (which lacks lipid-binding activity), supports this hypothesis.

We proposed previously that localization and ankyrin sensitivity of spectrin-PE/PC binding provides a support function of this site in ankyrin deficiencies [14]. In addition to the data provided in the present work, two further examples should be mentioned. In mouse erythrocytes with ankyrin deficiency, spectrin-based skeleton was still normally formed [24]. In a different work, Das et al. [40] showed that loss of the ankyrin-binding site in *Drosophila melanogaster* β-spectrin had only a mild effect either on the phenotype or on spectrin targeting to the membrane. Our results suggest that loss of the ankyrin-binding site may be compensated for by the presence of a PE-rich lipid-binding site that was still present in *Drosophila* β-spectrin. This part of the ankyrin-binding domain is one of the fragments among β-spectrin chains highly conserved in evolution (Fig. S5).

In conclusion, our data support the hypothesis that lipid binding by ankBDn is an important physiological feature of spectrin, which plays a role in maintaining the properties of membrane and membrane skeleton.

## Materials and Methods

### Constructs

We used a pRSET C expression vector carrying an ankyrin-binding domain [18] in mutagenesis PCR reactions. Constructs were based on a spectrin fragment consisting of 1684–1896 residues and mutations were introduced in the region 1768–1786. The following mutants were obtained by using Stratagene QuikChange Site-Directed Mutagenesis Kit: MutSSK (L1782S, L1785S, I1786K); MutSSD (L1782S, L1785S, I1786D); MutSSS (L1782S, L1785S, I1786S); WLSA (M1778S, W1779A); WSSA (L1775S, M1778S, W1779A); SSSA (W1771S, L1775S, M1778S, W1779A); SSSW (W1771S, L1775S, M1778S); CLSC (W1771C, M1778S, W1779C); and Ala8 (alanines at all positions from residues 1768 to 1775). For expression of ankSBD protein we used a previously obtained construct [31]. The constructs were maintained in *Escherichia coli* strain, XL1Blue. All mutated constructs were sequenced to verify mutations. Mutated spectrin fragment proteins were expressed in BL21(DE3)pLysS cells. Isopropyl β-D-thiogalactoside (100 mM) was used as an inducer for 3 h at 37°C. Cells were lysed and proteins were extracted with 8 M urea and 100 mM NaCl in 20 mM Tris-HCl, pH 8.0, and purified by immobilized Co^2+^-affinity chromatography (Clontech). Protein concentration was measured as described before [28]. The purity of His-tagged proteins was assessed on Coomassie Blue-stained SDS/PAGE (10%) gels (Fig. 2A).

### Circular dichroism

Circular dichroism (CD) measurements were performed on a JASCO J-715CD spectrometer as described previously [18]. Helical content was calculated from values of the amide nπ* transition at 222 nm ([θ_222_]), using a value of –36000 degrees·cm^2^·dmol^−1^ to represent 100% α helical content [41].

### Monolayer experiments

Monolayer measurements were performed by the Wilhelmy technique, using a Teflon trough (surface area 24 cm^2^) and a Nima tensiometer (NimaTechnology, Coventry, UK), at room temperature (21°C). Changes in surface pressure were measured after addition of protein into the subphase and are presented as *Δ*π. The measurements were taken after 25 min following the addition of the protein solution. For inhibition of lipid–protein interactions by ankyrin, expressed spectrin polypeptides were dialyzed against subphase buffer (5 mM Tris/HCl, pH 7.5, 0.5 mM EDTA, 150 mM NaCl, 0.5 mM dithiothreitol and 1 mM NaN_3_) and preincubated for 30 min at room temperature with purified recombinant spectrin-binding domain of ankyrin [31] at the desired polypeptide/ankyrin molar ratios, and were then injected into the subphase [17]. Curves were fitted according to the equation: B = B_min_+(B0-B_min_)/(1+10∧(Free-LogEC50)); B0 – binding when competitor concentration = 0; B_min_ – minimal binding level.

### Liposome-binding assays

#### Fluorescent labeling of proteins

Purified as described previously [15] erythrocyte spectrin and the expressed spectrin fragments were fluorescently labeled using tetramethylrhodamine-5-maleimide (Sigma Aldrich) according to the manufacturer's directions. Labeling efficiency, determined using a molar extinction coefficient of rhodamine of 80,000 at 543 nm, was ∼80%.

#### FRET-based assay

For FRET-based assay proteins were labeled as follows: 36 µl of 22.5 mM tetramethylrhodamine isothiocyanate (Molecular Probes) in DMSO was added to 1 ml of protein solution (∼1 mg/ml) in carbonate buffer, pH 9.2, in 5 ml portions upon stirring. The reaction was carried out overnight at 4°C in the dark. The unreacted label was quenched by adding Tris-HCl, pH 8.5, to a concentration of 50 mM and sucrose to 0.5 M, and the resulting mixture was dialyzed overnight to the “assay buffer” (20 mM Tris, pH 7.5, 150 mM NaCl). The labeling efficiency was ∼80%. PE/PC liposomes labeled with NBD-PE at a concentration of 0.5 mol% were prepared by hydration with the assay buffer. Next, the lipid vesicles were calibrated by extrusion through a 100-nm pore filter. Proteins labeled with TRITC were incubated with liposomes in the assay buffer for 30 min at room temperature (21°C). After incubation, NBD fluorescence was measured. K_D_ (equilibrium dissociation constant) and maximal binding capacity (B_max_) values were calculated according to the equation B = (B_max_ * Free)/(K_D_+Free) by using non-linear regression.

#### Electrochemiluminescence immunoassay

The EIA experiments were performed according to the manufacturer's protocol (Meso Scale Discovery, MSD^®^) based on the sandwich immunoassay which utilizes electrochemiluminescence to measure protein levels [42]. Briefly, liposomes were passively adsorbed on the electrode surface (1 h, 23°C), and the residual sites on the surface were blocked with 0.1% DB-blocker (1 h, 23°C). The surface was then washed three times with 20 mM TRIS-HCl, 150 mM NaCl and solutions containing the desired concentrations of proteins were added to each well. Binding was carried out for 2 h at 23°C. Wells were then washed and a solution of primary antibody (Qiagen) against the His-Tag sequence was applied (1 µg/ml, 23°C, 1 h). The wells were again washed, and secondary antibody (goat anti-mouse-STAG) was added (1 µg/ml, 23°C, 1 h). The wells were washed and reading buffer was added (MSD surfactant-free reading buffer). The background was determined from binding of both primary and secondary antibodies to liposomes. Data were acquired on a SECTOR Imager 6000. The recorded data were analyzed using GraphPad Prism 5.0 software using one site-specific binding algorithm. To measure the interactions between ankBDn and its mutants with the spectrin-binding domain (ankSBD) of ankyrin a previously published protocol was used [34]. K_D_ (equilibrium dissociation constant) and maximal binding capacity (B_max_) values were calculated according to the equation B = B_max_ * Free/(Free+K_D_)+nonspecific binding. In the inhibition assay, the equation B = (B0-B_min_) * exp(-Free * Free)+B_min_ was used to fit curves.

#### Pelleting assay

FAT liposomes were prepared by using 20 mM Tris, pH 7.5, containing 150 mM NaCl, and 20% dextran T-200000. The liposome suspension was diluted with the above buffer without dextran (assay buffer) and centrifuged at 30 000 g to remove small vesicles and untrapped dextran. Fluorescently labeled proteins were incubated with FAT liposomes in assay buffer for 30 min at room temperature (21°C). After incubation, the samples were centrifuged at 30 000 g for 20 min. The liposome pellets were dissolved in 1% (w/v) SDS for 10 min. Fluorescence measurements were performed using a Cary Eclipse Varian Spectrofluorimeter. The excitation and emission wavelengths for tetramethylrhodamine-labeled proteins were 541 and 575 nm respectively. Competition experiments were performed in the presence of an excess of unlabeled recombinant ankyrin-binding domain and its mutants, which were added to the samples with the liposomes and labeled spectrin. K_D_ (equilibrium dissociation constant) and maximal binding capacity (B_max_) values were calculated according to the equation B = (B_max_ * Free)/(K_D_+Free) by using non-linear regression. In the inhibition assay, the equation B = (B0–B_min_) * exp(-Free * Free)+B_min_ was used to fit curves.

### Preparation of resealed ghosts

Fresh blood samples were collected from healthy human volunteers (upon their informed, written consent) using anti-coagulant (0.8% citric acid monohydrate, 2.2% trisodium citrate, 2.2% glucose). Red cells were washed three times with 10 mM Tris-HCl buffer (pH 7.4) containing 120 mM KCl. Intact cells were lysed in ice-cold lysis buffer containing 0.6 mM MgATP in 5 mM Tris-HCl, 5 mM KCl, 1 mM MgCl_2_, pH 7.4 and centrifuged (15 000 g, 15 min, 4°C) and washed in the same buffer until pale pink. The ghosts were resuspended in resealing buffer (150 mM KCl, 1.6 mM MgCl_2_, 1 mM DTT, 0.6 mM MgATP, pH 7.4), and incubated for 60 min, at 37°C in the presence or absence of hemoglobin or recombinant ankBDn or its mutants (final concentration 1 mg/ml). After resealing, the suspension was centrifuged (10 000 g, 5 min, 4°C), and pellets and supernatants were collected for further analysis. Resealed ghosts were stained with lipophilic dye, Vybrant^®^ DiD (Molecular Probes), according to the manufacturer's instructions. The consent of the Ethics Committee was not necessary as the blood samples were taken in the clinical laboratory and used only for simple experiments on erythrocytes and no patient treatment or genetic analysis was involved.

### Transfection of HeLa cell culture

#### Transient expression of fluorescent recombinant proteins in HeLa cell culture

PCR products coding for ankBDn and its mutants (SSSA and CLSC) were cloned into pEGFP-C1 (Clontech). GFP was located at the N-termini of the constructs. HeLa cells (human epithelial carcinoma cell line) were transiently transfected in 24-well plates as described by Bok et al. [28]. HeLa cell line was obtained from Ludwik Hirszfeld Institute of Immunology and Experimental Therapy of Polish Academy of Sciences in Wrocław.

#### Immunostaining

Cells were washed several times with PBS and fixed with paraformaldehyde (2%) in PBS, permeabilized with 0.1% Triton X-100 in PBS, and washed again with PBS. Thereafter the cells were blocked for 15 min with 100% FBS and incubated for 1 h 45 min with primary antibodies (polyclonal rabbit anti-human Na^+^/K^+^ ATPase α1, Santa Cruz Biotechnology, 2 µg/ml and monoclonal mouse anti-human EEA1, BD Biosciences, 250 µg/ml) at room temperature. Cells were then washed three times with PBS. As a secondary antibody, TRITC-labeled goat anti-rabbit IgG (Jackson Immuno Research, 1∶100) or Cy-5 labeled donkey anti-mouse (BD Biosciences, 1∶100) was used. Cells were examined by confocal microscopy using a Zeiss LSM 510 Meta microscope and Plan Apo 63x/1.4 lens objective.

#### Western blot analysis

HeLa cells were collected 24 h after transfection and subjected to SDS-PAGE. Separated proteins were transferred to nitrocellulose membranes (Whatman). Antibodies and dilutions used included anti-ankBDn polyclonal antibodies prepared in our laboratory (1∶1000), and secondary antibody conjugated with alkaline phosphatase (Jackson ImmunoResearch) (1∶2500).

### Structure prediction and analysis

The spectrin model was built using homology modeling within Modeller 9v6 [43] from residues 1584 to 1889 choosing the structures of β-spectrin (PDB ID 3 kbt and 3edv) as templates. Residues 1764 to 1773 were defined as a 3_10_ helix [19] and modeled from an *ab initio* template of the sequence drawn in HyperChem.

The model of the spectrin structure was subjected to rigid minimization within Xplor-NIH [44] and restrained by the EPR-derived restraints [22] which had been collected in the presence of PE/PC liposomes. Distance restraints were implemented between Cα carbons of the residues. The three EPR-based distances used for the calculation were between residues 1699 and 1768 (20.5+/-2.5 Å), residues 1710 and 1779 (<25 Å), and residues 1750 and 1771 (19.4+/-2.5 Å).

Regions including residues 1584 to 1714, 1722 to 1760, and 1764 to 1889 were kept rigid during the minimization, allowing each helix to move freely and satisfy the distance restraints.

Mutant protein models used for analysis of hydrophobicity were modeled using the non-commercially available program 3D-Jigsaw from the website: http://bmm.cancerresearchuk.org/ ∼3djigsaw/ [45] and visualized by Yasara and 3D-mol open-source programs. The PDB ID code of the template structure is 3edu.

## Supporting Information

Figure S1 **Hydrophobic plot of 1768–1805 region.** A) WLSA, B) MutSSS, C) WSSA, D) SSSW , E) CLSC, F) Ala8, Deep red, hydrophobic, deep blue, hydrophilic; substitutions are marked.
(TIF)

Figure S2 **Binding of fluorescently labeled recombinant proteins by FAT PE/PC liposomes.** Amount of bound protein was calculated according to standard curve obtained for fluorescently labeled proteins. A) Comparison between ankBDn and quadruple mutant SSSA, using thermally denatured ankBDn as negative control. B) Binding curves obtained for the remaining mutants.
(TIF)

Figure S3 **SDS-PAGE analysis of pellets and supernatants collected after resealing erythrocyte ghosts.** P, pellet; S, supernatant. Arrow indicates resealed proteins. Similar amount of proteins was resealed in all cases and resealing efficiency was estimated to be 50–60%. The same volumes of pellet and supernatants were loaded onto the gel. Released spectrin is observed in supernatant collected from ghosts resealed with ankBDn. 10% polyacrylamide gel stained with Coomassie Blue.
(TIF)

Figure S4 **Analysis of observed aggregates.** A) Cells transfected with pEGFP reported plasmid (first row) and with ankBDn-GFP construct (second row) stained with anti-human EEA1 antibodies. No colocalization between aggregates and endosomes is observed. B) Western blot analysis with anti-ankBDn antibodies of lysed HeLa cells transfected with ankBDn-GFP or SSSA-GFP constructs. No substantial proteolysis is observed. Scale bars: 20 ìm.
(TIF)

Figure S5 **Alignment of known β-spectrin sequences corresponding to ankyrin-binding domain.** The box outlined in red indicates the 38 amino acid region encompassing the putative lipid-binding site. Arrows mark residues that were substituted in various ankBDn mutants.
(TIF)
