# Supplementary figures and images for: Key Amino Acid Residues of Ankyrin-Sensitive Phosphatidylethanolamine/Phosphatidylcholine-Lipid Binding Site of βI-Spectrin
Source: PLoS One. 2011 Jun 28;6(6):e21538. doi: 10.1371/journal.pone.0021538 (PMC3125217; doi:10.1371/journal.pone.0021538)

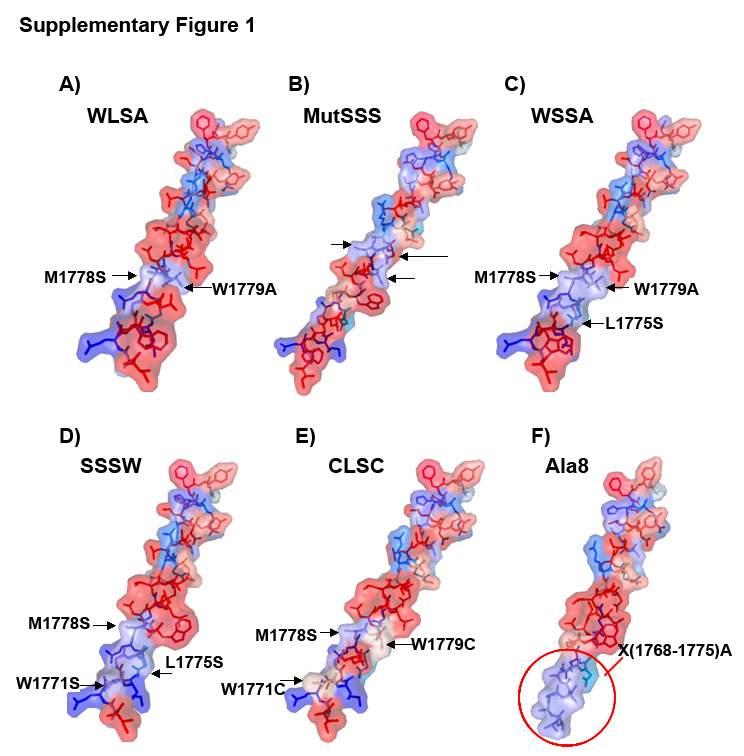

Supplement: Figure S1 — Hydrophobic plot of 1768–1805 region. A) WLSA, B) MutSSS, C) WSSA, D) SSSW , E) CLSC, F) Ala8, Deep red, hydrophobic, deep blue, hydrophilic; substitutions are marked. (TIF) [file pone.0021538.s001.tif]

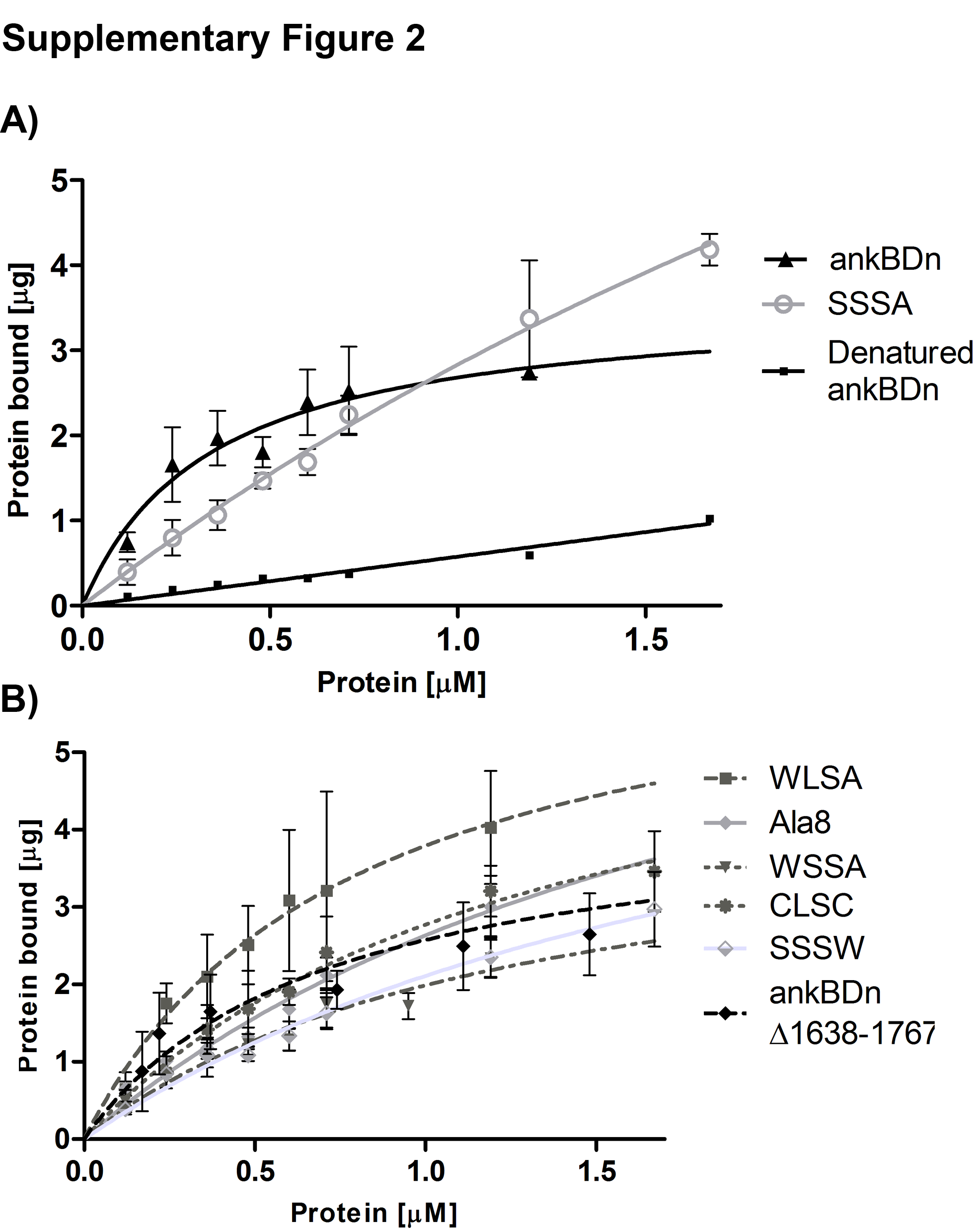

Supplement: Figure S2 — Binding of fluorescently labeled recombinant proteins by FAT PE/PC liposomes. Amount of bound protein was calculated according to standard curve obtained for fluorescently labeled proteins. A) Comparison between ankBDn and quadruple mutant SSSA, using thermally denatured ankBDn as negative control. B) Binding curves obtained for the remaining mutants. (TIF) [file pone.0021538.s002.tif]

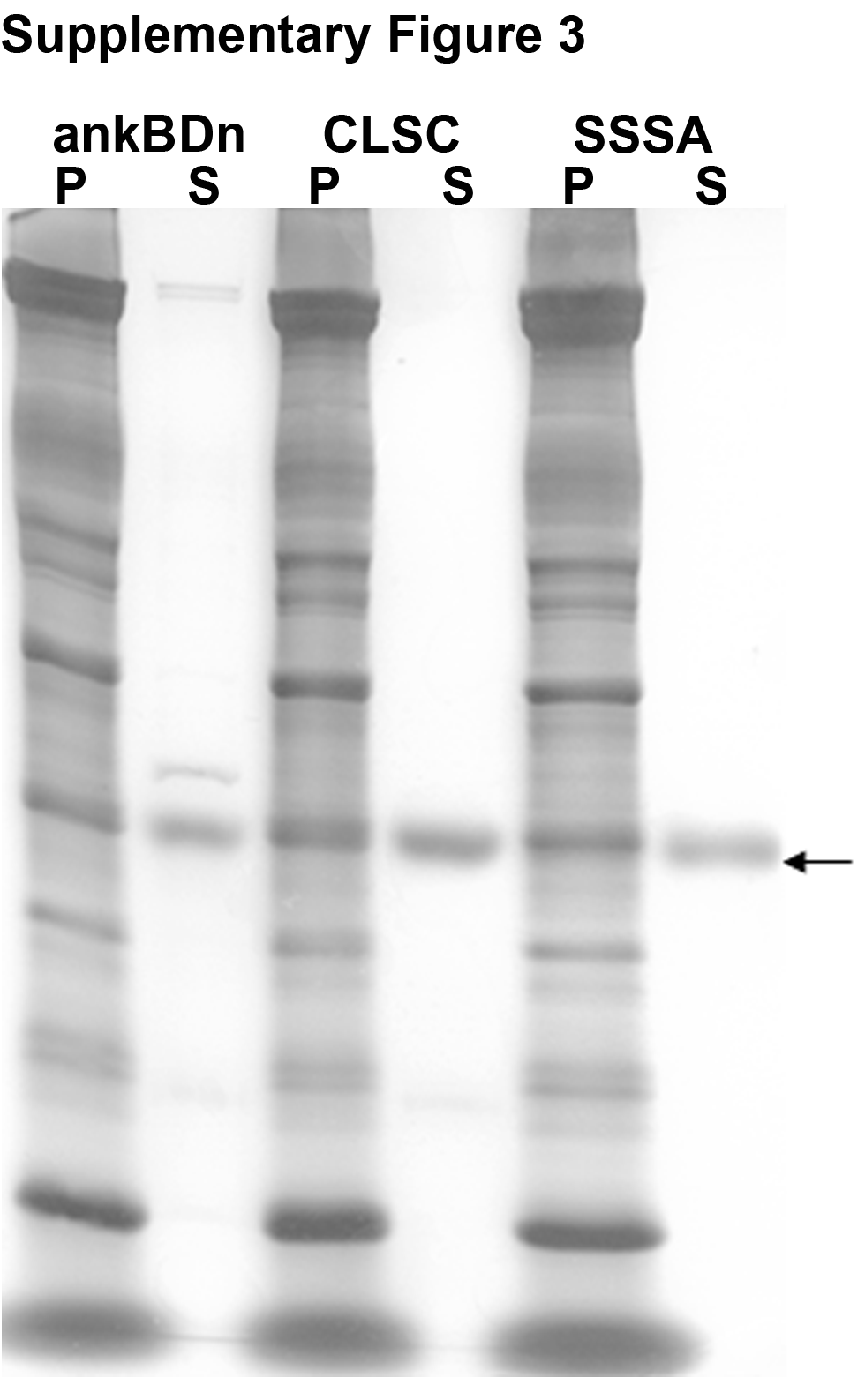

Supplement: Figure S3 — SDS-PAGE analysis of pellets and supernatants collected after resealing erythrocyte ghosts. P, pellet; S, supernatant. Arrow indicates resealed proteins. Similar amount of proteins was resealed in all cases and resealing efficiency was estimated to be 50–60%. The same volumes of pellet and supernatants were loaded onto the gel. Released spectrin is observed in supernatant collected from ghosts resealed with ankBDn. 10% polyacrylamide gel stained with Coomassie Blue. (TIF) [file pone.0021538.s003.tif]

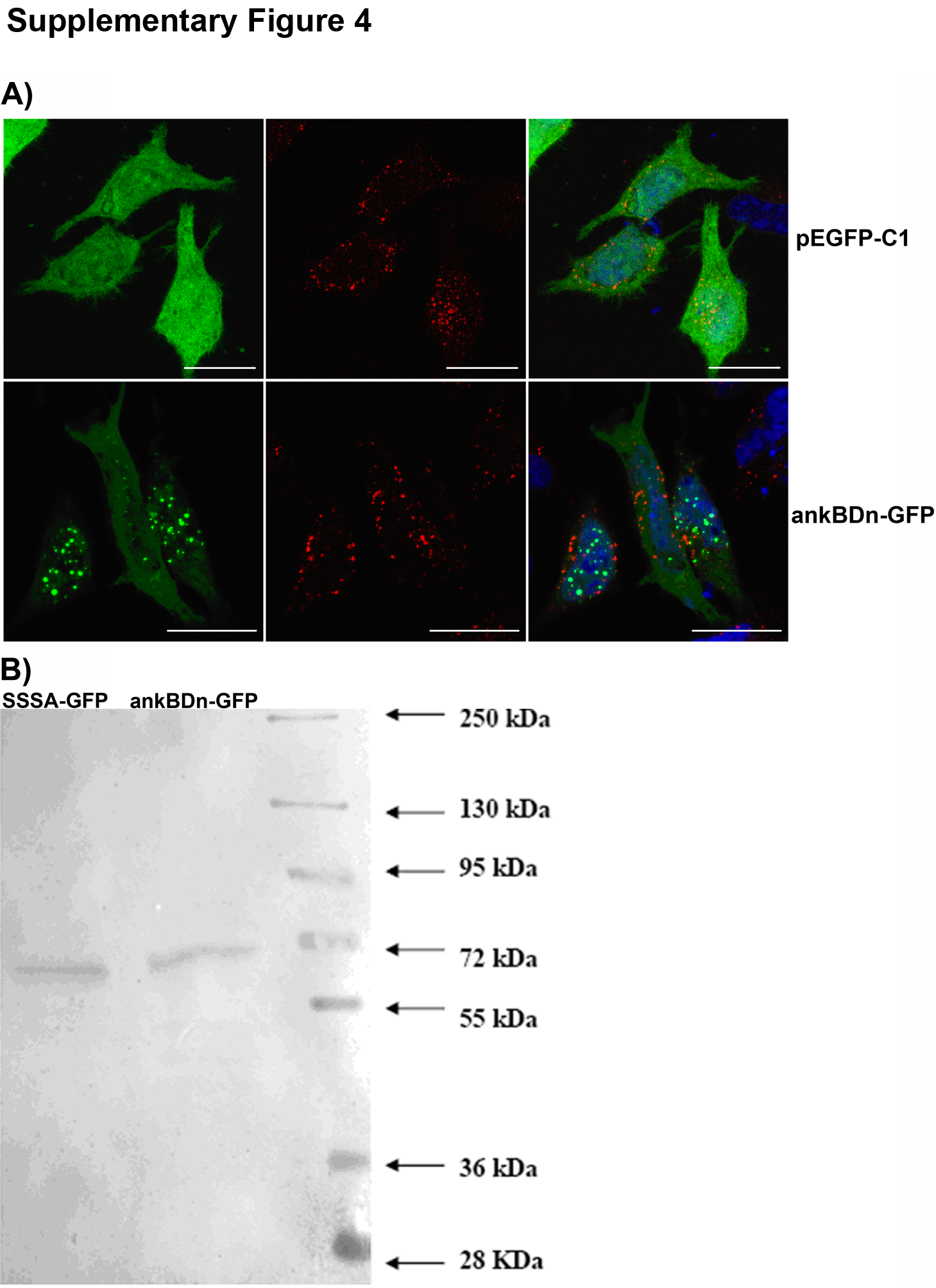

Supplement: Figure S4 — Analysis of observed aggregates. A) Cells transfected with pEGFP reported plasmid (first row) and with ankBDn-GFP construct (second row) stained with anti-human EEA1 antibodies. No colocalization between aggregates and endosomes is observed. B) Western blot analysis with anti-ankBDn antibodies of lysed HeLa cells transfected with ankBDn-GFP or SSSA-GFP constructs. No substantial proteolysis is observed. Scale bars: 20 ìm. (TIF) [file pone.0021538.s004.tif]

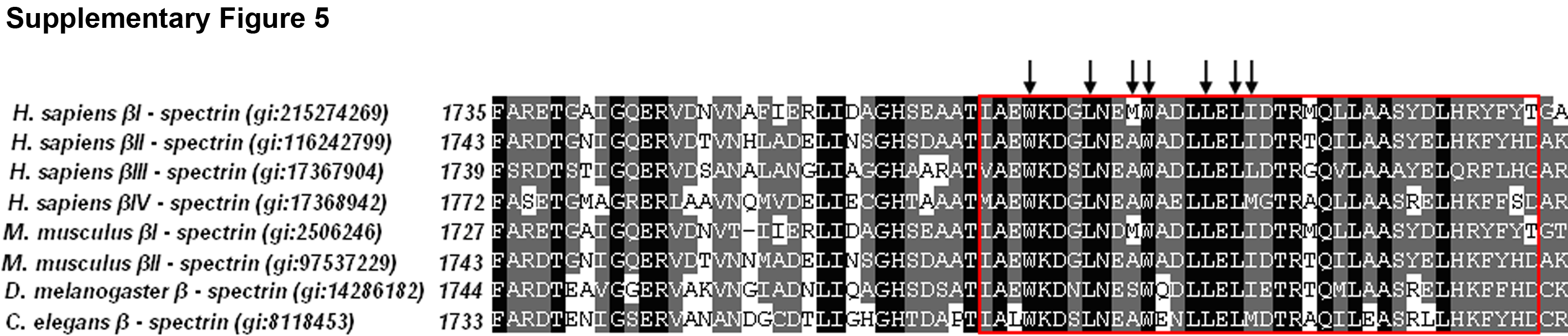

Supplement: Figure S5 — Alignment of known β-spectrin sequences corresponding to ankyrin-binding domain. The box outlined in red indicates the 38 amino acid region encompassing the putative lipid-binding site. Arrows mark residues that were substituted in various ankBDn mutants. (TIF) [file pone.0021538.s005.tif]
